# cGMP-dependent protein kinase Iα associates with the antidepressant-sensitive serotonin transporter and dictates rapid modulation of serotonin uptake

**DOI:** 10.1186/1756-6606-2-26

**Published:** 2009-08-05

**Authors:** Jennifer A Steiner, Ana Marin D Carneiro, Jane Wright, Heinrich JG Matthies, Harish C Prasad, Christian K Nicki, Wolfgang R Dostmann, Carrie C Buchanan, Jackie D Corbin, Sharron H Francis, Randy D Blakely

**Affiliations:** 1 Department of Pharmacology, Vanderbilt University School of Medicine, Nashville, TN 37232, USA; 2 Department of Molecular Physiology and Biophysics, Vanderbilt University School of Medicine, Nashville, TN 37232, USA; 3 Departments of Pharmacology and Molecular Physiology and Biophysics, University of Vermont, College of Medicine, Burlington, VT 05405, USA; 4 Department of Psychiatry, Vanderbilt University School of Medicine, Nashville, TN 37232, USA; 5 Center for Molecular Neuroscience, Vanderbilt University School of Medicine, Nashville, TN 37232, USA

## Abstract

**Background:**

The Na^+^/Cl^-^-dependent serotonin (5-hydroxytryptamine, 5-HT) transporter (SERT) is a critical element in neuronal 5-HT signaling, being responsible for the efficient elimination of 5-HT after release. SERTs are not only targets for exogenous addictive and therapeutic agents but also can be modulated by endogenous, receptor-linked signaling pathways. We have shown that neuronal A3 adenosine receptor activation leads to enhanced presynaptic 5-HT transport *in vitro *and an increased rate of SERT-mediated 5-HT clearance *in vivo*. SERT stimulation by A3 adenosine receptors derives from an elevation of cGMP and subsequent activation of both cGMP-dependent protein kinase (PKG) and p38 mitogen-activated protein kinase. PKG activators such as 8-Br-cGMP are known to lead to transporter phosphorylation, though how this modification supports SERT regulation is unclear.

**Results:**

In this report, we explore the kinase isoform specificity underlying the rapid stimulation of SERT activity by PKG activators. Using immortalized, rat serotonergic raphe neurons (RN46A) previously shown to support 8-Br-cGMP stimulation of SERT surface trafficking, we document expression of PKGI, and to a lower extent, PKGII. Quantitative analysis of staining profiles using permeabilized or nonpermeabilized conditions reveals that SERT colocalizes with PKGI in both intracellular and cell surface domains of RN46A cell bodies, and exhibits a more restricted, intracellular pattern of colocalization in neuritic processes. In the same cells, SERT demonstrates a lack of colocalization with PKGII in either intracellular or surface membranes. In keeping with the ability of the membrane permeant kinase inhibitor DT-2 to block 8-Br-cGMP stimulation of SERT, we found that DT-2 treatment eliminated cGMP-dependent kinase activity in PKGI-immunoreactive extracts resolved by liquid chromatography. Similarly, treatment of SERT-transfected HeLa cells with small interfering RNAs targeting endogenous PKGI eliminated 8-Br-cGMP-induced regulation of SERT activity. Co-immunoprecipitation studies show that, in transporter/kinase co-transfected cells, PKGIα specifically associates with hSERT.

**Conclusion:**

Our findings provide evidence of a physical and compartmentalized association between SERT and PKGIα that supports rapid, 8-Br-cGMP-induced regulation of SERT. We discuss a model wherein SERT-associated PKGIα supports sequentially the mobilization of intracellular transporter-containing vesicles, leading to enhanced surface expression, and the production of catalytic-modulatory SERT phosphorylation, leading to a maximal enhancement of 5-HT clearance capacity.

## Background

Signaling by the neurotransmitter serotonin (5-hydroxytryptamine, 5-HT) plays a critical, modulatory role in brain pathways supporting mood, appetite, sexual behavior, and reward. The inactivation of synaptic 5-HT is achieved largely through the actions of presynaptic serotonin transporters (SERT, SLC6A4). SERTs also participate in presynaptic 5-HT recycling to sustain neuronal 5-HT stores: this is most evident in studies with SERT KO mice that display a 60–80% reduction in brain 5-HT level [1]. These mice also display profound changes in 5-HT receptor coupling and a loss of psychostimulant (MDMA, "ecstasy") sensitivity [1-5], underscoring the importance of SERT for 5-HT signaling and drug response.

Increasing evidence indicates that SERT-mediated 5-HT clearance is controlled by multiple regulatory pathways that dictate both SERT plasma membrane expression and catalytic activity (reviewed in [6]). By analogy with the structure of the recently crystallized, SLC6 homolog LeuT_Aa _[7], SERTs are modeled to possess twelve transmembrane domains [8] with intracellular amino and carboxy termini. These latter domains possess multiple canonical Ser/Thr phosphorylation sites and are known to interact directly with other proteins, including Hic-5, neuronal nitric oxide synthase (nNOS), as well as heteromeric integrins that contain a β3 subunit [9-11]. Further specification and localization of the key molecules involved in SERT regulation, clarification of whether they form stable or transient associations with SERT, and evaluation of how loss of regulation contributes to SERT dysfunction may offer important insights into 5-HT-linked brain disorders such as depression, autism, and obsessive-compulsive disorder (OCD) [12-15]. In this regard, we and others have shown that multiple human SERT coding variants display compromised regulation through protein kinase C (PKC)-, cGMP-dependent protein kinase (PKG)-, and p38 mitogen-activated protein kinase (p38 MAPK)-linked pathways [16-19]. Among these coding variants are mutants found to associate with autism and OCD [20,21]. A detailed analysis of the signaling pathways that regulate SERT may thus make important contributions to our understanding of multiple neuropsychiatric disorders and offer new strategies for intervention.

Our previous work has elucidated the actions of multiple G-protein coupled receptors that can regulate SERT negatively (e.g. α2 adrenergic receptors [22]) or positively (e.g. A3 adenosine receptors (A3ARs, [23,24]). A3ARs enhance SERT trafficking and 5-HT clearance capacity as a consequence of elevations in intracellular Ca^++ ^and cGMP and PKG activation [23,24]. We have shown that PKG-dependent regulation of SERT by A3ARs is present both in cultured RBL-2H3 mast cells [23] and in serotonergic neuronal terminals monitored *ex vivo *or *in vivo *[24]. Consistent with these findings, SERTs are phosphorylated by treatment of cells or neurons with the general PKG activators 8-pCPT-cGMP and 8-Br-cGMP [17,25]. Two genes encoding homologous PKG proteins have been identified, PKGI and PKGII (also identified as cGK1 and 2, [26]), both of which can be stimulated by membrane-permeant cGMP analogs. PKGI is a soluble, cytosolic protein that is abundant in smooth muscle, platelets, and brain [27,28]. Mice lacking PKGI have profound smooth muscle deficits that lead to eventual death [29]. These deficits can be rescued in constitutive PKGI KO mice by smooth muscle-specific rescue using either of the two PKGI amino-terminal splice variants PKGIα or PKGIβ [30]. PKGII is a myristoylated, membrane-associated protein that is most abundant in intestine, brain, and kidney [31,32]. PKGII knockout mice exhibit dwarfism and defects in intestinal secretion [33]. Which of the two PKG genes participate in SERT regulation, or whether both contribute, is presently unknown though critical for further mechanistic studies that pursue whole animal genetic or pharmacological manipulations.

Neuroanatomical studies are lacking that document the precise localization in brain of PKG isoforms in relation to SERT expression, likely due to the insensitivity of available reagents for determining kinase localization *in situ *outside of very prominent sites of expression, such as cell bodies of the cerebellum (Steiner and Blakely, unpublished findings). However, we have demonstrated that A3 agonist stimulation of SERT in synaptosomes *in vitro *and in the hippocampus *in vivo *can be antagonized by the peptide DT-2, a membrane-permeant inhibitor proposed to block selectively PKGI [24,34]. In the present work, we demonstrate expression of DT-2 sensitive, cGMP-dependent kinase activity that comigrates with PKGI immunoreactive protein in RN46A cells, where we also document colocalization of SERT with PKGI, but not PKGII, by quantitative immunofluorescence approaches. SERT/PKGI colocalization is most evident when labeling of the transporter is achieved in permeabilized cells, consistent with a predominant role of PKGI in regulating of transporter vesicular trafficking. Consistent with this idea, PKGI-targeted siRNAs completely eliminate 8-Br-cGMP-triggered SERT stimulation, regulation that arises from elevated transporter surface expression. Co-immunoprecipitation studies reveal that SERT forms a stable complex with PKGI, with a greater efficiency of association achieved with PKGIα over PKGIβ. We discuss the impact of our findings with respect to a model for how intracellular SERT/PKGI associations support both trafficking-dependent and -independent modes of SERT regulation.

## Results

### Expression and colocalization of PKGI with SERT in RN46A cells

**Figure 1:**
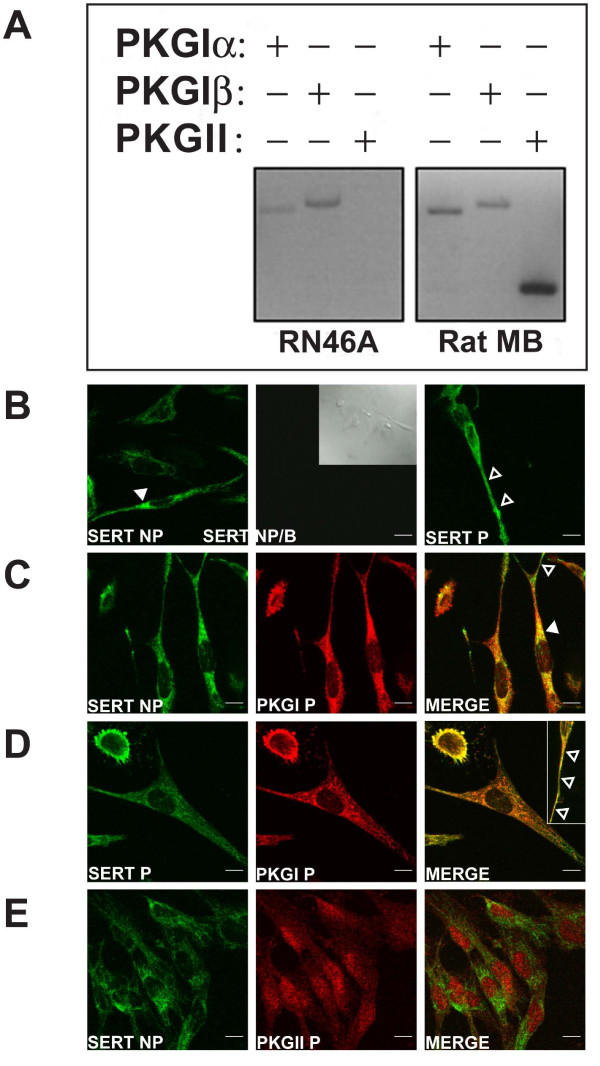
**Colocalization of SERT and PKGI in RN46A cells**. A) Total RNA from RN46A cells or rat midbrain was isolated and subjected to RT-PCR as described in Methods. Figure shows agarose gel analysis of cDNA products, revealing expression of PKGI but very little PKGII. B-E) Double immunocytochemical labeling of SERT, PKGI, and PKGII. RN46A cells were fixed, stained, and imaged by confocal microscopy as described in Methods. SERT staining was achieved with an ectodomain antibody (Advanced Targeting AB-N09). NP, nonpermeabilized. P, permeabilized by 0.2% NP40. NP/B, nonpermeabilized with antibody preabsorbtion by peptide (Advanced Targeting PR-03). DIC image is inset in B, middle, to demonstrate presence of cells. Inset image in D, right, shows staining in process. Closed arrows indicate perinuclear staining while open arrows indicate process staining. Scale bars represent 10 μm. Data are representative of at least three independent experiments.

To initiate studies of PKG gene products engaged in neuronal SERT regulation, we explored the expression of PKG isoforms in RN46A cells, a serotonergic cell line that we have previously found to display 8-Br-cGMP-stimulated SERT activity. Initially, we performed qualitatitive RT-PCR analysis on total RN46A RNA using oligonucleotide primers specific for PKGIα, PKGβ and PKGII. Compared to rat midbrain RNA, where we readily identified expression of all three gene products, RN46A RNA only yielded amplification of PKGIα and PKGIβ (Fig. 1A). Next, we pursued immunofluorescence studies of RN6A cells, where we confirmed readily detectable expression of PKGI and SERT (Fig. 1B–D) and low expression of PKGII (Fig 1E). Staining with PKGI or PKGII primary antibodies was eliminated with preabsorption against their respective antigens (Fig. 1B, middle panel; also see Additional File 1). Staining was also absent in cells treated with fluorescent secondary antibodies alone and in the case of PKGI, when cells were not permeabilized (data not shown). For SERT localization studies, RN46A cells were probed with a SERT ectodomain antibody under two conditions. One set of assays involved probing for SERT prior to permeabilization (NP, Fig. 1), followed by permeabilization of cells to detect cytoplasmic PKG. A second evaluation involved use of both SERT and PKG antibodies under permeabilized (P, Fig. 1) conditions. P cells displayed a broader cellular pattern of SERT staining than nonpermeabilized cells (compare Fig. 1B, left and right panels), consistent with detection of a pool of surface SERTs in NP cells. Dual-labeling experiments under these conditions revealed evidence of significant colocalization of PKGI with SERT under NP conditions, localized primarily to RN46A cell bodies (Fig. 1C, right panel, closed arrowhead). In these preparations, areas devoid of PKGI labeling, but positive for SERT, were clearly evident at the tips of processes (Fig. 1C, right panel, open arrowhead). With P conditions, PKGI/SERT colabeling was more extensive than under NP conditions, filling both the cell soma as well as processes (Fig. 1D, right panel). In keeping with a lack of detection of PKGII in the RN46A cells, low signal for PKGII was evident by immunofluorescence (Fig. 1E, middle panel) and was detected as a nuclear pattern surrounded by diffuse cytoplasmic labeling. SERT/PKGII colocalization evaluation in double labeling preparations (Fig. 1E, right panel) was quite distinct from that observed with PKGI, and showed no evidence for overlap in patterns of expression.

**Figure 2:**
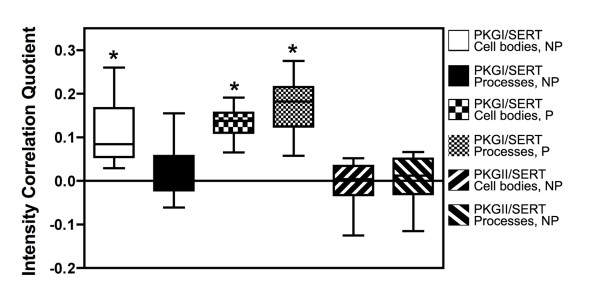
**Quantitative analysis of SERT/PKGI colocalization**. Cells were fixed and stained as shown representatively in Figure 1, and the Intensity Correlation Quotient was calculated for both cell bodies and processes under either nonpermeabilized (NP) or permeabilized (P) conditions, as described in Methods. Data represent mean values ± SEM from multiple cells in 4–6 fields, replicated in at least three experiments. Statistical analysis was conducted by one-sample *t *test. *, *p *< 0.001.

To assess PKG and SERT colocalization using quantitative approaches, we calculated the Intensity Colocalization Quotient (ICQ) under either NP or P conditions for both cell bodies and processes. As described by Li and colleagues [35], ICQ values represent a measure of the extent of correlation of intensity values in space for 2 separate fluorophores, where values range between -0.5 and 0.5 for negatively and positively correlated signals, respectively. As shown in Fig. 2, PKGI/SERT ICQ values fall significantly above 0 under some conditions, whereas PKGII/SERT analyses yield no ICQ values different from 0 under any condition, suggesting a random relationship of staining. For PKGI/SERT under NP conditions, significant colocalization is evident only in the cell body area. Under P conditions, significant colocalization of PKGI/SERT is evident for both the cell soma and processes, in keeping with the qualitative evaluations noted above. These findings provide quantitative evidence of PKGI/SERT colocalization (our PKGI antibody cannot distinguish PKGIα and β) and reveal that PKGI/SERT complexes are largely intracellular in distal processes.

### DT-2 sensitive PKGI activity in RN46A cells

**Figure 3:**
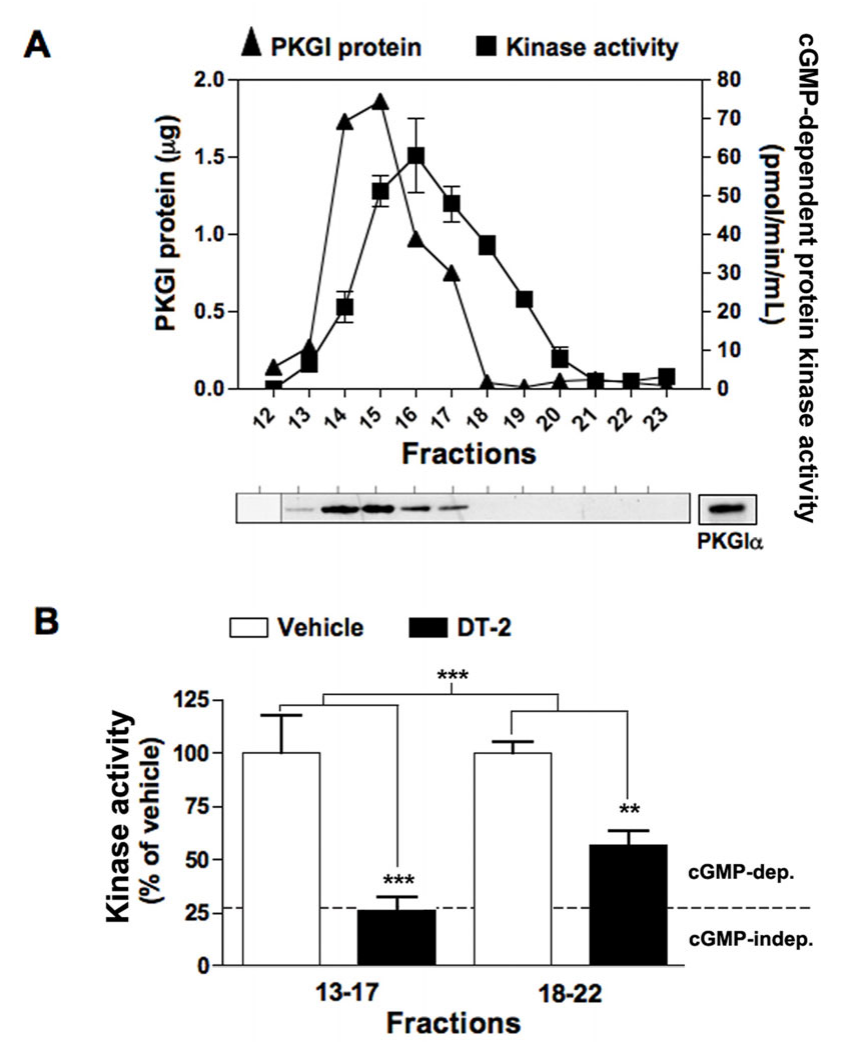
**DT-2 sensitive, PKGI activity in RN46A cells**. A) DEAE chromatography and phosphorylation assays of RN46A cell extracts were performed as described in Methods. Chromatogram displays cGMP-dependent (background-subtracted) kinase activity and quantitative protein measurement. cGMP-independent kinase activity was measured as 27.3% of total kinase activity. Representative immunoblot of fractions with detectable kinase activity is shown below chromatogram. B) Evaluation of DT-2 sensitivity in fractions containing detectable (13–17) or nondetectable (18–22) PKGI protein by immunoblot. Both fraction bins 13–17 and 18–22 displayed DT-2-sensitive kinase activity (***, *p *< 0.001; **, *p *< 0.01), with fractions overlaying PKGI protein being significantly more sensitive to DT-2 (***, *p *< 0.001). Dashed line represents cGMP-independent kinase activity equivalent to 27.3%. Data shown are from a single experiment, are representative of three independent experiments, and are presented as means ± SEM. Statistical significance was calculated via two-way ANOVA, followed by Bonferroni's post-test.

DT-2 is a membrane permeant peptide that was developed as a PKGI inhibitor [34] and has been previously demonstrated to eliminate SERT stimulation following A3AR stimulation or exogenous 8-Br-cGMP treatments [24,36]. Prior studies of kinase specificity *in vitro *using purified kinases, indicate a K_i _of 12.5 ± 3.0 nM for purified PKGI (either PKGIα or PKGIβ) versus 75.5 ± 18 μM for PKA [34]. We extended these findings to PKGII and PKCα, yielding K_i _values of 9.10 ± 1.3 μM and 26.6 ± 4.0 nM, respectively. Although DT-2 can inhibit PKCα at concentrations comparable to PKGI inhibition, PKC activation leads to SERT internalization rather than a movement to the surface and an enhancement of 5-HT uptake. Regardless, our findings add support to the contention that the DT-2 sensitivity of cGMP-dependent SERT regulation is evidence of PKGI versus PKGII involvement. To explore this issue further in the RN46A model, we fractionated RN46A cell lysates via DEAE-Sephacel chromatography, testing fractions for levels of PKGI-immunoreactive protein as well as levels of cGMP-dependent phosphorylation of the heptapeptide substrate (RKRSRAE). In Fig. 3A, we display a representative analysis, plotting both PKGI protein level and kinase activity from fractions where significant cGMP-dependent kinase activity was detected. Activity in our kinase assays does not necessarily derive from PKG activity as other kinases, notably PKA, can utilize cGMP for activation [37]. Thus, in Fig. 3B, we analyze the sensitivity of cGMP-stimulated kinase activity to DT-2. The fractions bearing the most significant level of PKGI protein (fractions 13–17; 93.9% of total PKGI immunoreactivity) exhibited a significantly greater degree of DT-2 sensitivity as compared to fractions where PKGI immunoreactivity was low or absent (fractions 18–22; 6.1%). Indeed, in fractions 13–17, all of the cGMP-stimulated kinase activity (Fig. 3B, dotted line) was eliminated whereas we detected DT-2 insensitive kinase activity in fractions 18–22. Together, these studies demonstrate the presence of DT-2-sensitive PKGI in RN46A cells and support the likelihood that DT-2 blockade of cGMP-dependent SERT stimulation in intact cells arises from antagonism of PKGI.

### PKGI is required for 8-Br-cGMP-mediated increases in SERT activity in transfected cells

**Figure 4:**
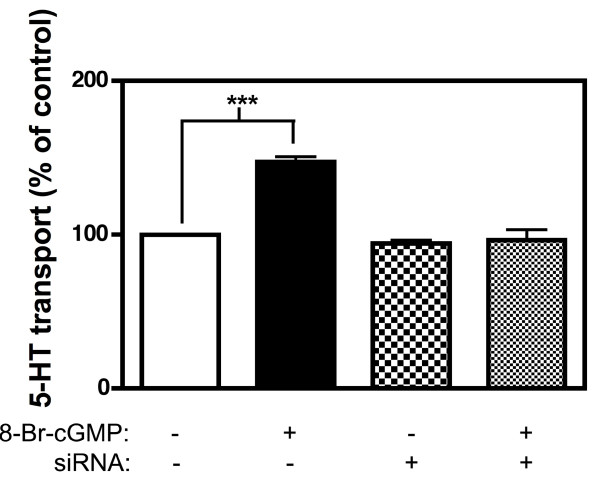
**Treatment of transfected HeLa cells with PKGI siRNA prevents 8-Br-cGMP-induced SERT stimulation**. Cells were transfected with hSERT and siRNA directed against human PKGI and assayed for 8-Br-cGMP stimulation of SERT activity as described in Methods. Upon 8-Br-cGMP stimulation, cells expressing hSERT (closed bar) demonstrate elevated 5-HT transport activity (147 ± 3.56%) absent from cells treated with PKGI siRNA (dotted bar, 96.4 ± 6.96%). Cells treated with siRNA in the absence of 8-Br-cGMP (checkered bar) show no change in 5-HT uptake (94.5 ± 1.88%) from control (open bar, 100 ± 0.010%). Each assay was repeated three times. Values are means ± SEM. ***, *p *< 0.001 versus control, calculated via one-way ANOVA followed by Tukey's post-test.

We have shown that SERT activity can be stimulated by 8-Br-cGMP in multiple heterologous expression systems, including transfected HeLa cells [17,20,36]. These observations allowed us to implement transient transfection of PKGI-directed siRNAs to assess whether evidence, independent of DT-2, could be gathered that PKGI is essential for 8-Br-cGMP triggered SERT stimulation. Mock or siRNA-treated HeLa cells (60 hrs) were subjected to either vehicle or 8-Br-cGMP treatment (100 μM, 10 min) followed by [^3^H]5-HT transport assays (20 nM, 10 min), as described in Methods. As shown in Fig. 4, PKGI siRNA treatments had no significant effect on basal SERT activity in either mock or siRNA transfections. Additionally, mock-transfected cells demonstrated the previously documented stimulation of SERT activity following 8-Br-cGMP treatment [17]. In contrast, cells treated with PKGI siRNA exhibited no 8-Br-cGMP-induced SERT stimulation.

### hSERT forms a stable association with PKGIα

**Figure 5:**
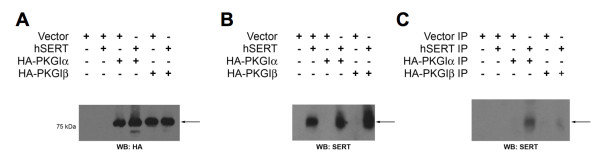
**hSERT forms a stable association with PKGIα**. hSERT and HA-PKGIα or HA-PKGIβ were coexpressed in HEK-293 T cells and coimmunoprecipitations were performed as described in Methods. Total cell extracts were blotted for A) HA to reveal PKG expression or B) SERT. C) Co-immunoprecipations of SERT and PKG using anti-HA for immunoprecipitations and anti-SERT for blotting. Experiment shown was replicated in four independent experiments with similar results.

Although our studies above implicate PKGI in SERT regulation, they do not address whether PKGI/SERT interactions are transient or stable, nor do they identify which isoform of PKGI is most likely involved in SERT regulation since our PKGI antibodies are not isoform specific. As SERT protein levels were too low in RN46A cells to permit co-immunoprecipitation of SERT/PKGI complexes, we performed transient co-transfections of HEK-293 T cells using His-tagged hSERT and HA-tagged PKGI isoforms. HA-PKGI complexes were isolated via anti-HA beads followed by immunoblotting of total and co-imminoprecipitated extracts using an antibody against SERT. Anti-HA blots of total cell lysates demonstrated equivalent expression of HA-PKGIα and HA-PKGIβ (Fig. 5A, arrow). Anti-SERT blots revealed equivalent expression of hSERT (Fig. 5B, arrow). We did note a broader, higher M_r _smear of SERT-immunoreactive species in lanes cotransfected with either PKG isoform. The origin of this altered SERT migration is as yet unclear, but could reflect a capacity of either isoform to trigger SERT phosphorylation or lead indirectly to the stabilization of multimeric, detergent-resistant SERT complexes. However, only anti-HA bead eluates of PKGIα revealed the presence of hSERT (Fig. 5C). These studies provide evidence that stable complexes of hSERT with PKGI are formed in transporter/kinase transfected cells and uncover an unexpected specificity for the PKGIα isoform in this model.

## Discussion

Increasingly, we appreciate that biogenic amine transporters typified by SERT are under complex regulatory control mediated by associated proteins and multiple signaling pathways [9-11,17,18,22,23,25,36,38-50]. Understanding the molecules and contingencies involved in SERT regulation is likely to enhance our abilities to manipulate SERT for therapeutic ends, as well as expand our understanding as to how SERT dysregulation contributes to mental illness [16,17,20,21]. Miller and Hoffman first identified a cGMP-linked pathway promoting rapid enhancement of SERT activity following adenosine receptor activation [44]. Thus, 8-Br-cGMP treatments recapitulate the actions of nonselective adenosine receptor agonists on RBL-2H3 cells (e.g. 5'-*N*-ethylcarboxamidoadenosine, NECA) and these authors hypothesized that cGMP-stimulated protein kinases might direct phosphorylation of SERT or a SERT-associated protein to trigger increased SERT activity. We now understand that A3ARs and/or 8-Br-cGMP treatments can increase SERT activity in multiple contexts, including the rat raphe cell line RN46A [36], transfected CHO and HeLa cells [17,18], rat midbrain and cortical synaptosomes *in vitro *and the mouse hippocampus *in vivo *[47]. In all cases, pharmacological inhibition of PKG blocks stimulation of SERT activity. Furthermore, biochemical and surface expression studies reveal that PKG activators lead to both SERT phosphorylation [17,18,47] and enhanced SERT insertion into the plasma membrane [17,19,23], providing an opportunity for steady-state increases in 5-HT uptake capacity. SERT trafficking regulation leads to p38 MAPK activation that we have shown shifts the transporter to a higher affinity state for 5-HT recognition [36]. Progress has also been achieved in identifying a number of SERT-interacting proteins [9-11,43,49] whose associations can impact both SERT trafficking and transport rates. In this report, we provide evidence that PKGIα is responsible for rapid, 8-Br-cGMP-dependent, DT-2-sensitive SERT stimulation. Moreover, we demonstrate that mammalian cells can organize a stable association of PKGIα with SERT, an interaction likely to support the enhanced surface trafficking of the transporter evident after treatment of cells with PKG activators.

In RN46A cells, we established using qualitative and quantitative approaches that endogenous PKGI and SERT colocalize. Although PKGII could not be detected by RT-PCR, low levels of PKGII immunoreactivity were evident via confocal-assisted immunofluorescence studies. However, no evidence for SERT/PKGII colocalization was evident. Interestingly, SERT/PKGI colocalization was nonuniform, being most prominent in permeabilized preparations and absent in the distal segments of nonpermeabilized cells. One possible explanation for these findings is that SERT recruits PKGI to vesicles as part of a coordinated mechanism to achieve regulated plasma membrane trafficking and that, in distal processes, SERT moves to the plasma membrane after dissociation of PKGI. Extensive studies to date in our lab have failed to document consistent interactions between PKGI and SERT (Steiner and Blakely, unpublished findings), indicating a role for an intermediate in formation of SERT/PKGIα complexes. This intermediate could result in a SERT (or PKG) post-translational modification or represent a SERT-associated protein lacking under the conditions of *in vitro *incubations of purified proteins. Additional studies are clearly needed to elaborate the manner by which transporter/kinase associations are formed. Future studies should also explore dynamic features of the SERT/PKGI association, such as whether these interactions change upon vesicle membrane fusion and localization of SERT to plasma membrane subdomains.

PKG activation has been found to alter actin dynamics and vesicular trafficking [51,52] as well as promote phosphorylation of a single Thr (Thr276) on SERT whose mutation can eliminate SERT stimulation [47]. Whether Thr276 is phosphorylated directly by PKG, or possibly by p38 MAPK, which PKG is known to activate [23,36,53], is unknown. Since Thr276 is positioned within a short intracellular loop connecting transmembrane domains proposed to control 5-HT exit from the transporter [18,54], it seems most likely that this site has relevance for catalytic modulation, which we have shown is governed by p38 MAPK [23,36]. PKGI might then contribute to mobilization of SERT vesicles not by phosphorylation of SERT, but by phosphorylation of SERT vesicle tethering proteins. However, it is also possible that additional phosphorylation sites targeted by PKGI may exist; these could support transporter trafficking but be too transient to be captured by standard biochemical methods. In addition to the PKGIα associations we have defined, SERT associates with the phosphatase PP2A [43]. The specific sites in SERT that bind, or are dephosphorylated by PP2A, are not known, but a polymorphism (Gly56Ala) within the hSERT amino terminus leads to constitutive phosphorylation that cannot be further elevated by 8-Br-cGMP. Moreover, the Gly56Ala is a catalytically activated variant [17,19] that also displays diminished sensitivity to the PP2A specific inhibitor fostriecin [19]. Thus, it is possible that the SERT/PKGIα associations documented here are mutually exclusive with, or are modulated by SERT/PP2A interactions and that these two enzymes govern phosphorylation sites linked to SERT trafficking, as well as catalytic modulation. Interestingly, p38 MAPK is known to phosphorylate and activate PP2A [55], providing a path by which PKGI activation could lead to both p38 MAPK-dependent transporter catalytic activation and efficient dephosphorylation of trafficking regulatory SERT sites. A mirror image of this idea related to SERT inactivation and internalization has been proposed involving PKC and PP2A by our lab [40,43] and the work of Ramamoorthy's group [41]. Future studies that elucidate the sites targeted by PP2A and PKC should greatly clarify these ideas. Since OCD and autism-associated SERT variants appear to impact PKG, p38 MAPK and PP2A-dependent transporter regulation [18], this effort may also have significant impact on our understanding of the origins of 5-HT linked brain disorders.

We have recently proposed by analogy to the classical picture of insulin-dependent glucose transporter regulation [56] that two modes of SERT trafficking to the plasma membrane exist: one, a finite capacity, regulated pathway overseen by PKG, p38 MAPK, PP2A and PKC, and a second, constitutive pathway that is relatively insensitive to these proteins but which can be more greatly populated by heterologous overexpression. RN46A cells endogenously express a low level of SERT and display substantial elevations (≥ 200%) in SERT activity following 8-Br-cGMP treatment or p38 MAPK stimulation [36]. High-level heterologous expression of SERT in transfected cells, in our hands, leads to SERT proteins that demonstrate greatly reduced, and in some cases, no cGMP-dependent regulation. Due to this characteristic, we routinely constrain SERT expression in transfected cells where SERT regulation is being assessed, including the HeLa model we have used in this study. Our goal is to approach the low level of SERT expression observed in RN46A and RBL-2H3 cells. Unfortunately, such low expression levels make biochemical studies, such as SERT/PKGI co-immunoprecipitation experiments, extremely challenging, if not impossible. Although we have utilized the higher expressing HEK-293T model for our co-immunoprecipitation studies, our goal here was not to parallel functional studies, but rather to determine whether a portion of PKGI can assemble in a SERT complex and whether PKGI isoform specificity is evident. Additionally, we expect the pool of PKGI that associates with SERT to be small, and likely differentially localized, compared to the total PKGI pool. This possibility likely explains why we do not observe ICQ values for SERT and PKGI approaching 0.5, the theoretical maximum, even in permeabilized cells. Our blots of siRNA-treated HeLa extracts demonstrate only a modest reduction in total PKGI level [see Additional file 2] despite full elimination of 8-Br-cGMP triggered SERT stimulation. We also observed a full elimination of p38 MAPK-dependent regulation of SERT after only a modest reduction in p38α MAPK level by p38α MAPK-targeted siRNA treatment [36]. Since siRNAs are cotransfected with hSERT to evaluate transporter regulation, we thought it important to determine whether the full inhibition of regulation could be due to full suppression of kinase expression, though limited to the small percentage of cells transfected. Indeed, when we determined the percentage of cells transfected with siRNA using toxic siRNAs [see Additional file 2], we found a good match to the percent reduction in PKGI protein (~15% in both cases).

## Conclusion

We provide evidence that native SERT and PKGI are coexpressed in rat serotonergic RN46A cells where the two proteins colocalize, particularly when intracellular transporter pools are visualized *in situ *using permeabilized cells. In these cells, we corroborate the utility of the membrane-permeant peptide inhibitor DT-2 to eliminate cGMP-stimulated kinase activity, sensitivity that co-migrates with immunoreactive PKGI following ion exchange chromatography. Using PKGI siRNA, we establish a critical role for endogenous PKGI in 8-Br-cGMP triggered SERT regulation. We demonstrate that stable SERT/PKGIα complexes can be isolated from kinase/transporter co-transfected cells, revealing an unexpected isoform specificity that will propel future studies to evaluate how the alternatively spliced amino-terminal sequences that distinguish between PKGIα and β support PKGIα associations. Our studies thus illuminate a key facet of the molecular network that underlies rapid regulation of SERT via cell signaling pathways and compel further investigation of the mechanisms by which PKGIα/SERT associations are modulated by antidepressants, drugs of abuse, and disease-associated SERT mutations.

## Methods

### Reagents

Most chemicals were purchased from Sigma-Aldrich (St. Louis, MO). Culture medium (Dulbecco's modified Eagle's medium and Ham's F-12) was prepared by the Vanderbilt Media Core using Invitrogen (Invitrogen Corporation, Carlsbad, CA) reagents. Trypsin-EDTA, glutamine, and penicillin/streptomycin were also purchased from Invitrogen. Fetal bovine serum was obtained from Atlanta Biologicals (Norcross, GA). Anti-HA High Affinity Matrix beads and rat anti-HA monoclonal antibody were purchased from Roche (Basel, Switzerland). H8 hydrochloride was obtained from Cayman Chemical (Ann Arbor, MI). 5-hydroxy [^3^H]tryptamine trifluoroacetate ([^3^H]5-HT, 76 Ci/mmol) was purchased from Amersham Biosciences Inc. (Piscataway, NJ). Rabbit polyclonal anti-PKGI antibodies were a generous gift from Dr. Robert Feil (University of Tübingen, Tübingen, Germany) or were purchased from Assay Design, Inc. (Ann Arbor, MI). Rabbit polyclonal antibody directed against PKGII was kindly provided by Dr. Hugo De Jonge (Erasmus University, The Netherlands). Mouse SERT monoclonal antibody was from Advanced Targeting Systems (San Diego, CA).

### Cell culture and transfection

RN46A cells (a generous gift from Dr. Scott Whittemore, University of Louisville, Louisville, KY) were maintained in DMEM/F-12 (1:1 in volume) containing 250 mg/L G418, 10% FBS, 2 m M L-glutamine, 0.1 U/mL penicillin-0.11 g/mL streptomycin. HEK-293 T cells and HeLa cells were cultured in DMEM, 10% FBS, 2 m M L-glutamine, 0.1 U/mL penicillin-0.11 g/mL streptomycin. For co-expression studies, HEK-293 T cells were plated at a density of 100,000 cells/mL in individual wells of 6-well plates one day before transfection. Transfections were performed with *Trans*It^®^-LT1 (Mirus Bio Corporation, Madison, WI) and 500 ng of each cDNA, in accordance with manufacturer's instructions. For the siRNA studies, HeLa cells were plated at a density of 10,000 cells/mL in individual wells of a 24-well plate. Transfections were performed with Lipofectamine™ 2000 (Invitrogen), 25 ng pcDNA3-hSERT, and 100 n M siRNAs. For the siRNA studies, pcDNA3-hSERT and ON-TARGETplus SMARTpool human PRKG1 siRNAs (Thermo Fisher Scientific, Pittsburgh, PA) were used for transfections. To evaluate siRNA transfection efficiency, Allstars Hs Cell Death Control siRNAs (Qiagen, Valencia, CA) were transfected as described for PKG siRNAs as described by the manufacturer and dying/dead cells counted as a proportion of the total number of cells transfected by phase microsocopy.

### Plasmid constructs

The pcDNA3-His-hSERT construct was generated from previously described pcDNA3-hSERT [39] by adding a [His]_6_-Gly epitope sequence after the ATG start site using the QuikChange site-directed mutagenesis kit (Stratagene, La Jolla, CA). pcDNA3-HA-PKGIα and pcDNA3-HA-PKGIβ were kindly provided by Dr. Bo Cen (Columbia University, New York, NY).

### RT-PCR Studies

Total RNA from adult male, Sprague-Dawley rat midbrain and from RN46As was isolated using 5 Prime PerfectPure RNA kits (Fisher Scientific, Pittsburgh, PA) as described in the manufacturer's protocol. 1 μg of RNA was subjected to reverse transcription followed by PCR (RT-PCR) using the TITANIUM™ One-Step RT-PCR Kit (Clontech Laboratories, Inc., Mountain View, CA) and PKGIα (forward-5' GGTACATACAATCCACAATCTC 3', reverse-5' CCTCTCCTCTCCTTCCTTTTAG 3'), PKGIβ(forward-5' GGTACATACAATCCACAATCTC 3', reverse-5' AATCTGCACACTGAAACTCTCG 3') and PKGII (forward-5' GATGTTCACCGCAAGACCT 3', reverse-5' CGATGTAACTGCCCTGTTGATA 3') specific oligonucleotide primers (Invitrogen Corporation, Carlsbad, CA). Reaction products were resolved by 2% agarose gel electrophoresis and sequenced using dideoxy terminator sequencing (Vanderbilt University Medical Center DNA Sequencing Core).

### Immunostaining

The immunofluorescence protocol was adapted from previous work [57]. Briefly, 1–2 days before fixation, RN46A cells were seeded onto circular poly-D-lysine-coated coverslips in 24-well plates. Cells were fixed with 4% paraformaldehyde for 30 min., followed by either permeabilization with 0.2% NP40 or application of primary antibodies overnight at 4°C without detergent (nonpermeabilized conditions). Treatment with secondary antibodies conjugated to fluorophores Cy2 or Cy5 for 1 hr was followed by mounting of coverslips to charged slides. Indirect immunofluorescence images were captured using an LSM510 confocal microscope (Vanderbilt University Medical Center Cell Imaging Shared Resource supported by National Institutes of Health grants CA68485, DK20593, DK58404, HD15052, DK59637 and EY08126). Images were quantified using the Integration Colocalization Analysis section of WCIF Image J . Intensity Correlation Quotient (ICQ) values are presented and were taken as evidence for dependent staining or colocalization when the ICQ value falls between 0 and + 0.5. Independent staining or lack of colocalization was indicated when ICQ values were not different from 0 or were between 0 and -0.5. Data were analyzed by one-sample t-test comparing means versus a value of 0.

### Kinase inhibition assays using DT-2

Kinetic assays (K_i_, IC-50) were performed as previously described [34,58] with purified recombinant kinases and multiple concentrations of DT-2 (YGRKKRRQRRRPP-LRKKKKKH). Recombinant PKG Iα, PKG Iβ, PKA Iα (catalytic subunit) and PKGII were expressed from insect cell cultures using the Bacmid baculovirus expression system (Gibco/Invitrogen) and purified to apparent homogeneity as described recently [34]. Recombinant PKCα (human) was obtained from Calbiochem. All enzymes were assayed using saturating concentrations of the peptide TQAKRKKSLAMA as substrate. PKG isoforms were activated with 1 μM cGMP, PKA was stimulated with 1 μM cAMP, and PKC was activated using 50 μg/μL phospholipids and 100 ng/mL 12-O-Tetradecanoylphorbol-13-acetate.

### Fractionation and activity assays

Approximately 500 million RN46A cells were homogenized in ice-cold 10 mM potassium phosphate pH 6.8, 1 mM EDTA, and 25 mM β-mercaptoethanol (KPEM) and centrifuged at 10,000 × *g *for 20 min. at 4°C. The supernatant was applied to a pre-equilibrated 0.9 × 8 cm DEAE-Sephacel column and the column was washed with KPEM with 20 mM NaCl. A linear gradient (20 mM to 280 mM NaCl) was next run on the column and 30-drop fractions were collected. As previously described [59], kinase assays to determine PKG activity in each fraction were conducted in the presence and absence of 10 μM cGMP (a PKG activator) and 136 μg/mL heptapeptide substrate (RKRSRAE) as the PKG substrate. The cAMP-dependent protein kinase (PKA) inhibitor PKI-tide (5–24) was also included in the assay in order to block PKA activity. Activity was expressed as picomoles of [^32^P] incorporated per min per mL of sample.

### Co-immunoprecipitations and immunoblotting

Two days post-transfection with his-hSERT and HA-PKGI plasmids, HEK-293 T cells were detergent-extracted with 1% TRITON-X-100 and prepared for immunoprecipitation, as previously described [43]. Briefly, protein concentrations of lysates were determined with a bicinchoninic acid assay kit (Pierce, Rockford, IL), using bovine serum albumin as a standard. 50–100 μg total protein was immunoprecipitated with anti-HA High Affinity beads (Roche) for 1 hr and eluted with Laemmli sample buffer. Samples were subjected to SDS polyacrylamide gel electrophoresis and electroblotted to polyvinylidene difluoride membrane (Amersham), followed by incubation with primary antibodies. Blots were thoroughly washed with PBS containing 0.5% Tween-20 and developed with enhanced chemiluminescence (Amersham) with multiple exposures captured to ensure nonsaturation conditions.

### Transport assays

60 hrs after transfection with pcDNA3-hSERT and siRNAs, HeLa cells were subjected to 8-Br-cGMP treatment and 5-HT transport assays as previously described [17]. Briefly, transfected cells were treated with 100 μM 8-Br-cGMP for 10 min., then incubated with 20 n M [^3^H]5-HT for 10 min. After thorough washing with Krebs-Ringers-Hepes, pH 7.4, cells were solubilized in MicroScint 20 (PerkinElmer, Waltham, MA) and [^3^H]5-HT accumulation was quantified using a TopCount plate scintillation counter (Perkin Elmer). Specific 5-HT uptake was determined by subtracting the amount of [^3^H]5-HT taken up by nontransfected cells.

### Statistical analyses

All data were derived from experiments replicated a minimum of three times. Values are expressed as mean ± SEM. All Statistical analyses were completed in GraphPad Prism (GraphPad, San Diego, CA). In Fig. 2, one-sample Student's *t *tests were performed by comparing mean values to 0, and a value of *p *< 0.05 was taken as statistically significant. Analysis by one-way or two-way ANOVA was used followed by post-hoc testing (Bonferroni or Tukey) for Fig. 3 and 4, and a value of *p *< 0.05 was accepted as statistically significant. In Additional file 2, Analysis via a Repeated Measures One-Way ANOVA was utilized followed by Bonferroni post-tests, and a value of *p *< 0.05 was accepted as statistically significant.

## Competing interests

The authors declare that they have no competing interests.

## Authors' contributions

JAS designed and carried out experiments and data analysis resulting in Figures 1, 2, 3, 4, 5, and drafted the manuscript. AMDC provided direction in experimental design, data analysis and interpretation, and paper layout/editing. JW generated DNA constructs and provided assistance with immunohistochemical studies. HJGM provided training and guidance with experiments resulting in Figure 2. HCP completed experiments resulting in Figure 4. CKN and WRD performed kinase specificity assays noted in Results. JDC and SHF oversaw and participated in experiments comprising Figure 3 and assisted in data interpretation. RDB designed and coordinated the full study and assisted in manuscript construction. All authors read and approved the final manuscript.

## Supplementary Material

Additional file 1 **PKGI antibody specifically labels RN46A cells**. The data provided demonstrate the specificity of the PKG antibody. **Additional Figure 1. PKGI antibody specifically labels RN46A cells**. RN46A cells were fixed, permeabilized, stained, and imaged by confocal microscopy as described in Methods. Left panel) RN46A cells stained with anti-PKGI (Assay Designs, KAP-PK002E). Right panel) Antibody preabsorption with bovine lung PKGIα blocks the majority of PKGI staining, indicating antibody specificity. DIC image is inset in right panel to demonstrate presence of cells. Scale bars represent 10 μm. Data are representative of at least three independent experiments. Click here for file

Additional file 2 **siRNA knockdown of PKGI due to low transfection efficiency**. The data provided support the results shown in Figure 4 by demonstrating analysis of the siRNA-mediated protein knockdown and corresponding transfection efficiency. **Additional Figure 2. siRNA knockdown of PKGI due to low transfection efficiency**. HeLa cells were plated identically to those used for Figure 4, and assayed for PKGI protein and cell viability as described in Methods. A) Knockdown of PKGI protein by siRNAs. Transfected and siRNA-treated HeLa cells were lysed and analyzed via immunoblot for PKGI and β-actin immunoreactivity (IR). Analysis via a Repeated Measures One-Way ANOVA (control, mock, PKGI siRNA) of raw values for PKGI abundance normalized for β-actin signal in the same sample indicates a significant overall effect of PKGI siRNA (*p *< 0.05), with Bonferroni post-tests indicating no significant difference between mock and control but a significant (* = *p *< 0.05) difference between PKGI siRNA and control. Representative blot image is shown below quantitation. Percent changes are plotted for ease of evaluation. B) Measurement of transfection efficiency. The AllStars Hs cell death control siRNA (cell death siRNA; QIAGEN Inc., Valencia, CA) was used to quantitate siRNA transfection efficiency in the HeLa cells 48 hrs. post-transfection. Data are representative of at least three independent experiments, and results are presented as means ± SEM. Click here for file
