# Supplementary figures and images for: cGMP-dependent protein kinase Iα associates with the antidepressant-sensitive serotonin transporter and dictates rapid modulation of serotonin uptake
Source: Mol Brain. 2009 Aug 5;2:26. doi: 10.1186/1756-6606-2-26 (PMC2731736; doi:10.1186/1756-6606-2-26)

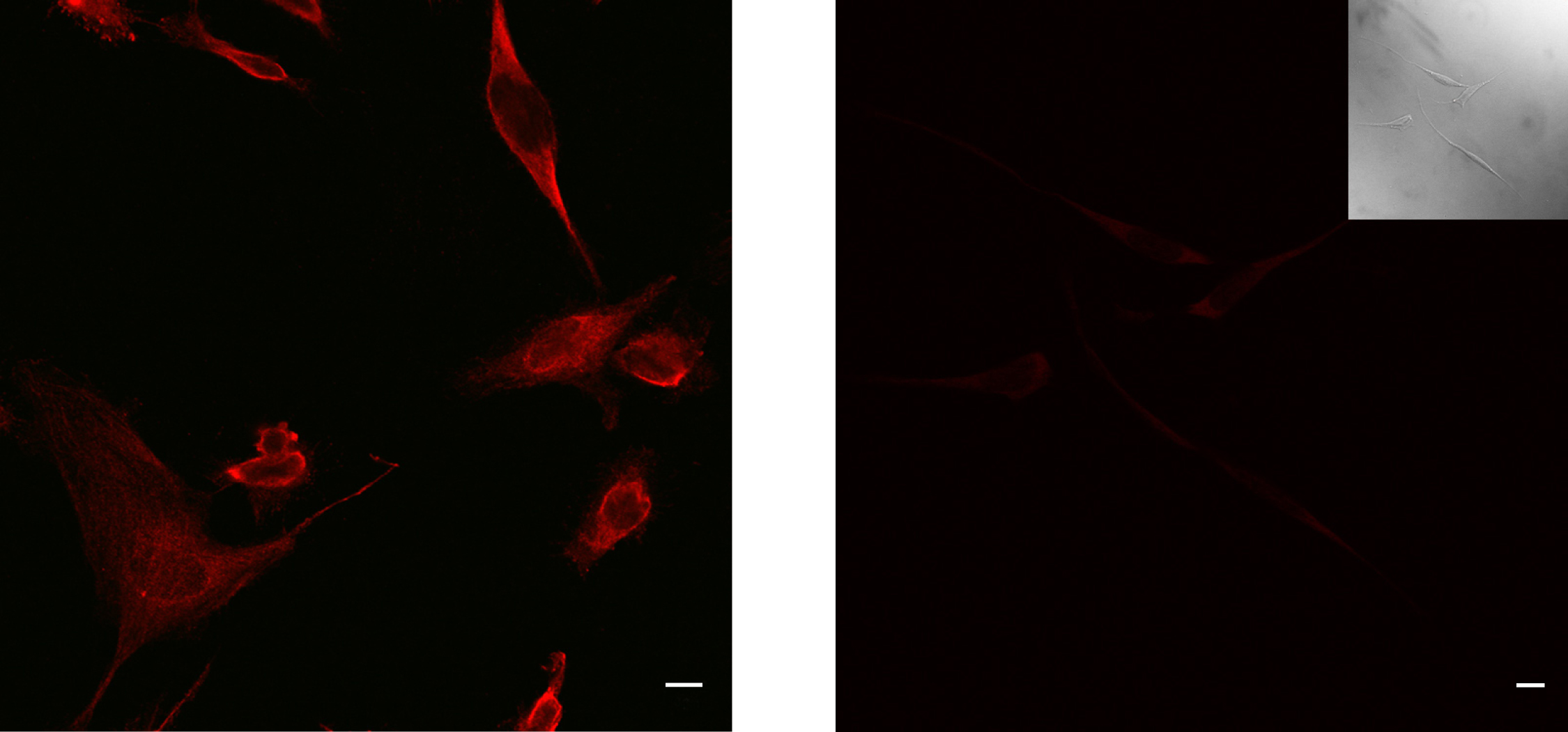

Supplement: Additional file 1 — PKGI antibody specifically labels RN46A cells. The data provided demonstrate the specificity of the PKG antibody. Additional Figure 1. PKGI antibody specifically labels RN46A cells. RN46A cells were fixed, permeabilized, stained, and imaged by confocal microscopy as described in Methods. Left panel) RN46A cells stained with anti-PKGI (Assay Designs, KAP-PK002E). Right panel) Antibody preabsorption with bovine lung PKGIα blocks the majority of PKGI staining, indicating antibody specificity. DIC image is inset in right panel to demonstrate presence of cells. Scale bars represent 10 μm. Data are representative of at least three independent experiments. [file 1756-6606-2-26-S1.jpeg]

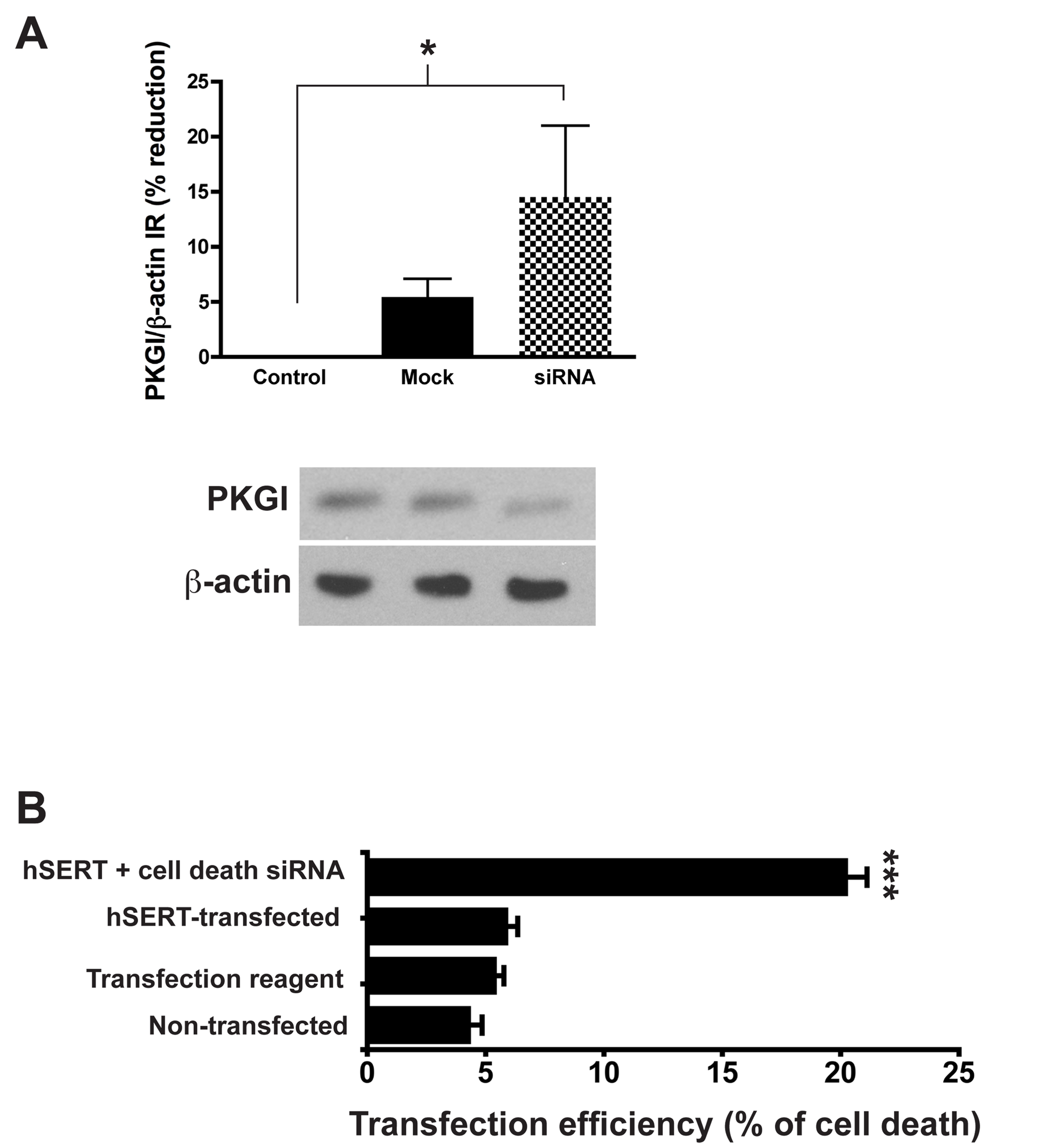

Supplement: Additional file 2 — siRNA knockdown of PKGI due to low transfection efficiency. The data provided support the results shown in Figure 4 by demonstrating analysis of the siRNA-mediated protein knockdown and corresponding transfection efficiency. Additional Figure 2. siRNA knockdown of PKGI due to low transfection efficiency. HeLa cells were plated identically to those used for Figure 4, and assayed for PKGI protein and cell viability as described in Methods. A) Knockdown of PKGI protein by siRNAs. Transfected and siRNA-treated HeLa cells were lysed and analyzed via immunoblot for PKGI and β-actin immunoreactivity (IR). Analysis via a Repeated Measures One-Way ANOVA (control, mock, PKGI siRNA) of raw values for PKGI abundance normalized for β-actin signal in the same sample indicates a significant overall effect of PKGI siRNA (p < 0.05), with Bonferroni post-tests indicating no significant difference between mock and control but a significant (* = p < 0.05) difference between PKGI siRNA and control. Representative blot image is shown below quantitation. Percent changes are plotted for ease of evaluation. B) Measurement of transfection efficiency. The AllStars Hs cell death control siRNA (cell death siRNA; QIAGEN Inc., Valencia, CA) was used to quantitate siRNA transfection efficiency in the HeLa cells 48 hrs. post-transfection. Data are representative of at least three independent experiments, and results are presented as means ± SEM. [file 1756-6606-2-26-S2.jpeg]
